# The Ameliorative Effects of a Tocotrienol-Rich Fraction on the AGE-RAGE Axis and Hypertension in High-Fat-Diet-Fed Rats with Metabolic Syndrome

**DOI:** 10.3390/nu9090984

**Published:** 2017-09-07

**Authors:** Hong Sheng Cheng, So Ha Ton, Joash Ban Lee Tan, Khalid Abdul Kadir

**Affiliations:** 1 School of Science, Monash University Malaysia, Bandar Sunway, Selangor 46150, Malaysia; ton.soha2016@gmail.com (S.H.T.); tan.ban.lee@monash.edu (J.B.L.T.); 2 School of Medicine and Health Sciences, Monash University Malaysia, Bandar Sunway, Selangor 46150, Malaysia; khalid.kadir@monash.edu

**Keywords:** advanced glycation end product, cholesterol, hepatic steatosis, myeloperoxidase, peroxisome proliferator-activated receptor, vitamin E

## Abstract

The clinical value of tocotrienols is increasingly appreciated because of the unique therapeutic effects that are not shared by tocopherols. However, their effect on metabolic syndrome is not well-established. This study aimed to investigate the effects of a tocotrienol-rich fraction (TRF) from palm oil in high-fat-diet-treated rats. Male, post-weaning Sprague Dawley rats were provided high-fat (60% kcal) diet for eight weeks followed by a TRF (60 mg/kg) treatment for another four weeks. Physical, metabolic, and histological changes were compared to those on control and high-fat diets respectively. High-fat feeding for eight weeks induced all hallmarks of metabolic syndrome. The TRF reversed systolic and diastolic hypertension, hypercholesterolemia, hepatic steatosis, impaired antioxidant defense, and myeloperoxidase hyperactivity triggered by the high-fat diet. It also conferred an inhibitory effect on protein glycation to reduce glycated hemoglobin A1c and advanced glycation end products (AGE). This was accompanied by the suppression of the receptor for advanced glycation end product (RAGE) expression in the liver. The treatment effects on visceral adiposity, glycemic control, triglyceride level, as well as peroxisome proliferator-activated receptor α and γ expression were negligible. To conclude, treatment with a TRF exhibited protective effects on the cardiovascular and liver health in addition to the amelioration of plasma redox imbalance and AGE-RAGE activation. Further investigation as a therapy for metabolic syndrome is therefore worthwhile.

## 1. Introduction

Being an important micronutrient and a powerful lipophilic antioxidant, vitamin E has received extensive research attention in the aspects of anti-inflammation, anti-cancer, chronic metabolic disorders, and neurodegenerative diseases ever since its discovery about a century ago [1,2,3]. Fundamentally, vitamin E can be divided into two major classes: tocopherols and tocotrienols, each with α, β, γ, and δ subtypes. Tocotrienols are differentiated from tocopherols by the presence of an unsaturated isoprenoid side chain with three double bonds. As α-tocopherol was the first natural form of vitamin E identified, it drew most of the research attention, rendering other isoforms, most notably tocotrienols, poorly understood.

Nevertheless, in recent years, the paradigm of vitamin E research has been shifting, whereby studies about tocotrienols are increasingly emphasized. In fact, many studies have reported that tocotrienols have similar antioxidant capacity [4] and superior anti-cancer properties in comparison to tocopherols [5,6]. More importantly, tocotrienols also exhibit cholesterol-lowering [7], neuroprotective [8], and anti-aging [9] effects, which are not shared by tocopherols. The bioactivities of tocotrienols are summarized comprehensively in several review articles [10,11,12]. Several promising natural sources in which tocotrienols can be found in high abundance include oil palm, barley, and rice. The tocotrienol/tocopherol ratios are 1:1, 1.9:1, to 3:1 for rice bran oil, barley, and palm oil, respectively [11]. Therefore, identifying the medicinal uses of tocotrienols may create added value to these cultivated plants, which subsequently could be translated into economic growth.

On the other hand, given that elevated oxidative stress, low-grade systemic inflammation, and deranged lipid and glucose homeostasis are well-implicated in the pathogenesis of metabolic disorders like obesity, metabolic syndrome, type 2 diabetes mellitus, and cardiovascular disease, the known bioactivities of tocotrienols make it an appealing therapeutic candidate. Indeed, many recent studies have confirmed that treatment with tocotrienols or a tocotrienol-rich fraction (TRF) effectively ameliorated glucose dysregulation, hypercholesterolemia, hypertension, oxidative stress, as well as proinflammatory response in animal studies [13,14,15,16,17]. Despite the promising pre-clinical results, only the cholesterol-lowering and antioxidant effects have been consistently demonstrated in human studies [18,19,20,21,22], whereas other health benefits to humans like antihypertensive and antihyperglycemic activities remain inconclusive [21]. 

In this context, most of the bioactivities of tocotrienols are highly indicated in metabolic syndrome, but our pre-existing knowledge is established primarily based on diabetic animal models or hypercholesterolemic patients. Hypothetically, according to the known activities, tocotrienols or a TRF could potentially be a useful treatment of metabolic syndrome. Nonetheless, research on this aspect is rather limited. Considering the ever-increasing global prevalence of obesity and metabolic syndrome and promising value of tocotrienols, exploring the therapeutic effects and associated underlying mechanism of tocotrienols in obesity and metabolic syndrome is pertinent. Taken together, the present study aimed to examine the metabolic effects of tocotrienols in high-fat-diet-induced obese rats. The findings are discussed with reference to the physiological, biochemical, histopathological, and transcriptional alterations. 

## 2. Materials and Methods

### 2.1. Animal Ethics and Housing Conditions

The use and handling of animals in the present study have been approved by Monash University Monash Animal Research Platform Animal Ethics Committees (AEC approval No.: MARP/2015/060) in compliance to the Australian Code of Practice for the Care and Use of Animals for Scientific Purposes outlined by National Health and Medical Research Council. Male, post-weaning (3-week-old) Sprague Dawley rats of 45–70 g were obtained from Monash University Malaysia Animal Facility. The rats were kept at 23 ± 1 °C with a 12 h light/dark cycle and given ad libitum access to homemade purified ingredient-based diets and tap water throughout the entire experiment. 

### 2.2. Experimental Design and Treatment

Twenty-one post-weaning rats were randomized into three groups (*n* = 7 per group), namely control diet, high-fat diet, and TRF groups. Control diet group was treated with a low-fat (10% kcal) diet, while the high-fat diet and TRF groups were given a high-fat diet (60% kcal) for 8 weeks to induce metabolic syndrome. The diets were formulated based on AIN-93G diet [23], and their compositions are shown in Table 1.

**Figure 1:**
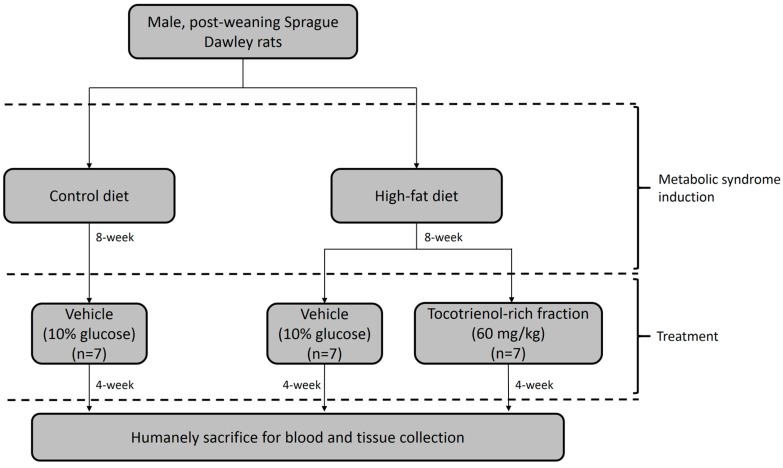
Experimental design of the present study.

After 8 weeks, the TRF group were treated with a 60 mg/kg commercially available TRF from palm oil known as Tocovid SupraBio^TM^ (Hovid, Ipoh, Malaysia) via oral gavage. The TRF is composed of 23.5% (*w*/*w*) α-tocotrienol, 43.2% γ-tocotrienol, 9.8% δ-tocotrienol, and 23.5% α-tocopherol. The dosage was selected based on a previous work [24]. The TRF was suspended in a 10% (*w*/*v*) glucose solution to minimize animal resistance to the administration procedure [25]. Control and high-fat diets groups were given the vehicle (a 10% glucose solution) via the same administration route. The treatment duration was 4 weeks, during which the rats were fed with the pre-assigned diets. The experimental design is illustrated in Figure 1.

The diets and water were refilled daily. Body weight, food, and water intake were also measured every day. At the end of the experiment, the rats were subjected to 12 h fasting prior to euthanasia with carbon dioxide. Blood samples were collected from the posterior vena cava and transferred into tubes with 0.5 M EDTA to avoid coagulation. The samples were then centrifuged immediately at 4 °C, 2000× *g* for 20 min to obtain the plasma specimens, which were snap-frozen in liquid nitrogen and stored at −80 °C until use. The liver, kidney, and retroperitoneal white adipose tissue (rWAT) were excised, washed with phosphate buffered saline (PBS), and weighed. Half of the liver and rWAT was snap-frozen and stored at −80 °C for RNA extraction and gene expression study, while the other half was stored in 10% (*v*/*v*) neutral buffered formalin for tissue fixation and histology. 

### 2.3. Blood Pressure Measurement

Systolic and diastolic blood pressure was measured with Mouse and Rat Tail Cuff Blood Pressure System (IITC Life Sciences, Los Angeles, CA, USA). The rats were placed into a plastic restrainer one at a time to restrict their movement throughout the measurement. A tail-cuff with a pulse transducer was applied onto the tail of the restrained rats. The rat was then placed into a well-ventilated chamber equilibrated at 32 °C for 15–20 min to facilitate the dilatation of caudal arteries. Next, the triplicate readings of the systolic and diastolic blood pressure were recorded. The procedure was performed once per week.

### 2.4. Biochemical Assays

Fasting blood glucose was determined with Accu-Chek^®^ Performa glucometer (Roche Diagnostics, Indianapolis, IN, USA), while fasting plasma insulin was measured using Mercodia Ultrasensitive Rat Insulin ELISA (Mercodia, Uppsala, Sweden). Homeostasis model assessment of the β-cell function (HOMA %β) and insulin sensitivity (HOMA %S) were calculated based on the fasting glucose and insulin levels [26] and expressed as a percentage of the control diet group. Glycated Hemoglobin A1c (HbA1c) and advanced glycation end products (AGEs) were quantified with the Rat Hemoglobin A1c (HbA1c) kit (Crystal Chem, Downers Grove, IL, USA) and the OxiSelect^TM^ Advanced Glycation End Product (AGE) Competitive ELISA kit (Cell Biolabs, San Diego, CA, USA), respectively. 

The lipid profile, including the triglyceride, total cholesterol (TC), and non-esterified fatty acid (NEFA) were measured with Randox TR1607 Triglycerides, CH200 Cholesterol, and FA115 Non-Esterified Fatty Acids kits (Randox, Dublin, UK). Chylomicron, low-density lipoprotein (LDL), and very low-density lipoprotein (VLDL) were precipitated from the plasma using the Randox CH203 HDL-cholesterol Precipitant kit (Randox, Dublin, UK), and the supernatant was subjected to the CH200 Cholesterol kit for the determination of high-density lipoprotein (HDL) cholesterol. Non-HDL-cholesterol was calculated by subtracting HDL cholesterol from total cholesterol.

Oxidative markers such as total plasma antioxidant capacity and myeloperoxidase activity were also measured with the OxiSelect^TM^ ORAC Activity Assay kit and the OxiSelect^TM^ Myeloperoxidase Chlorination Activity Assay kit (Cell Biolabs, San Diego, CA, USA) respectively. All analysis with the commercial kits was conducted in duplicates according to the manufacturers’ instructions.

### 2.5. Oral Glucose Tolerance Test (OGTT)

OGTT was carried out before and after the treatment in Weeks 8 and 12. Food was suspended for 8 h prior to the measurement of fasting blood glucose level. This was the initial reading at 0 min. Then, they were given a glucose load of 2 g/kg as a 40% (*w*/*v*) glucose solution via oral gavage. Blood glucose levels were measured as 30, 60, 90, and 120 min after administration of the glucose load. 

### 2.6. Plasma Electrolyte Levels

Atomic absorption spectrophotometry was used for the determination of plasma electrolyte levels. Plasma specimens were diluted by 500 and 50 times with distilled water for the measurement of sodium and potassium levels, respectively. The concentration of the electrolytes were determined with PerkinElmer Atomic Absorption Spectrophotometer Analyst 100 (PerkinElmer, Waltham, MA, USA) using a sodium/potassium hollow cathode lamp. The wavelength was set at 589 nm and 766 nm for measuring sodium and potassium concentrations, respectively. The actual concentration of the electrolytes was calculated based on a standard curve.

### 2.7. Tissue Processing and Histology

Conventional tissue processing, which includes dehydration, clearing, and infiltration of the liver and rWAT specimens with paraffin wax, was performed following formalin fixation. The tissues were then embedded in paraffin wax and stored at 4 °C. Thin sections (5 μm) were produced and stained with hematoxylin and eosin (H&E) to visualize the morphology of the tissues. Nikon Eclipse TS100 (Nikon, Tokyo, Japan) was used to capture the microscopic images of the tissues. ImageJ was used to measure the adipocyte size of the rWAT based on the method published by Parlee et al. (2014) [27].

### 2.8. Hepatic Lipid Extraction

Total lipid extraction of the liver tissues was carried out based on the Folch method [28]. Briefly, 150 mg of the snap-frozen liver tissues were ground into powder and homogenized in 20 mL of chloroform/methanol (2:1). The homogenates were vortexed for 1 min and sonicated for 20 min. This was followed by centrifugation at 1000× *g* for 10 min. The supernatant was washed with 0.2 volume of water, vortexed for 1 min, and centrifuged at 1000× *g* for 5 min. The upper fraction was discarded. The remaining fraction was rinsed with 1 mL of methanol/water (1:1) and centrifuged at 1000× *g* for 5 min. The upper fraction was removed, while the lower chloroform fraction that contained the total lipids was dried with a rotary evaporator. The hepatic total lipid extracts were weighed and then reconstituted in 1 mL of 1% (*w*/*v*) bovine serum albumin and subjected to a triglyceride assay with Randox TR1607 Triglycerides (Randox, Dublin, UK). 

### 2.9. RNA Extraction and cDNA Synthesis

Total RNA of the rWAT was isolated with Tri-RNA reagent (Favorgen, Ping-Tung, Taiwan) followed by the Qiagen RNeasy Mini kit (Qiagen, Hilden, Germany) to clean up the RNA sample, while that of the liver was extracted with Qiagen RNeasy Mini kit directly. The concentration and purity of the RNA were determined by measuring the absorbance at 260 nm and 280 nm with Infinite^®^ 200 PRO (TECAN, Zürich, Switzerland). RNA integrity was examined with agarose gel electrophoresis to check 18 S and 28 S ribosomal RNA. RNase-free DNase I (ThermoFisher Scientific, Waltham, MA, USA) treatment was performed prior to cDNA synthesis, which was performed with the Qiagen Omniscript Reverse Transcription Kit (Qiagen, Hilden, Germany).

### 2.10. Quantitative PCR (qPCR)

Rotor-Gene Q (Qiagen, Hilden, Germany) was used to perform SYBR green-based qPCR of receptor for advanced glycation end product (RAGE), soluble RAGE (sRAGE) peroxisome proliferator-activated receptors (PPARs) α and γ in the liver and rWAT. Hypoxanthine phosphoribosyltransferase 1 (HPRT1), succinate dehydrogenase complex flavoprotein subunit A (SDHA), and β-actin (BAC), which demonstrate stable expression in the target tissues, were selected as the endogenous reference genes for normalization of the genes of interest [29]. The nucleotide sequences of the forward and reverse primers as well as the accession numbers are outlined in Table 2. PCR conditions are shown in Table S1. Normalized Ct/ΔCt values of the genes of interest were calculated using the following formula:

ΔCt = average of Ct _*reference genes*_ − Ct _*gene of interest*_ (1)

### 2.11. Statistical Analysis

Statistical analysis was performed with Statistical Package for the Social Sciences (SPSS) 22.0. Dependent variables with repeated measures such as the cumulative weight gain, food and calorie intake, blood pressure and pre- and post-treatment OGTT were analyzed with a mixed model ANOVA using “time” as the within-subjects factor and “treatment group” as the between-subjects factor. The pairwise comparisons were performed with Bonferroni correction. Other variables were analyzed with one-way ANOVA followed by Tukey’s test. The level of statistical significance was pre-set at *p* ≤ 0.05.

## 3. Results

### 3.1. Weight Gain and Adiposity

**Figure 2:**
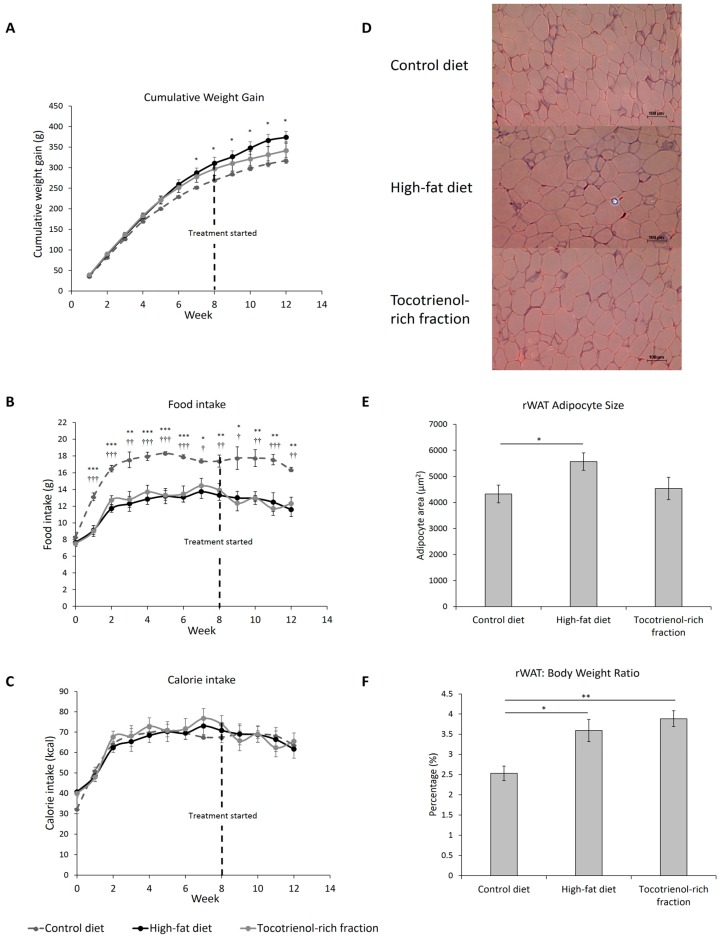
Cumulative weight gain (**A**), food intake (**B**), and calorie intake (**C**) of the rats assigned to different treatment groups. A tocotrienol-rich fraction was administered after 8 weeks of high-fat feeding, as indicated by the black dotted line. The representative images of the H&E-stained retroperitoneal white adipose tissues (×100 magnification) of each group are shown (**D**) and the average adipocyte areas are illustrated in bar plot (**E**). Retroperitoneal white adipose tissue weight-to-body weight ratio (**F**) was measured and expressed in percentage. Error bars indicate SEM. Sample size was *n* = 7 per group. For Figure 2A–C, *, **, *** indicate *p* < 0.05, 0.01, 0.001 between control and high-fat diet groups; †, ††, ††† indicate *p* < 0.05, 0.01, 0.001 between control diet and tocotrienol-rich fraction groups. For Figure 2E,F, * Indicates *p* < 0.05 and ** Indicates *p* < 0.01 between groups. rWAT: retroperitoneal white adipose tissue.

Figure 2A shows that the consumption of a high-fat diet, compared to the control diet, caused increased weight gain from Week 6 to the end of the experiment, while the TRF did not improve the weight control. The food intake of high-fat-diet- and TRF-treated rats was lower than that of the control diet group (Figure 2B). This was to compensate for the higher energy content of the high-fat diet, which explains the comparable calorie intake between all groups (Figure 2C).

High-fat diet increased the area of visceral adipocytes by more than 28%—a condition known as adipocyte hypertrophy (Figure 2D,E). Even though the TRF seems to reduce the adipocyte size, the difference did not reach statistical significance. The treatment also did not reduce the rWAT depot, which was otherwise increased by high-fat feeding by about 42% (Figure 2F). As such, high-fat diet led to the onset of central obesity and visceral adiposity, while treatment with the TRF has a limited effect on the condition.

### 3.2. Blood Pressure and Electrolyte

**Figure 3:**
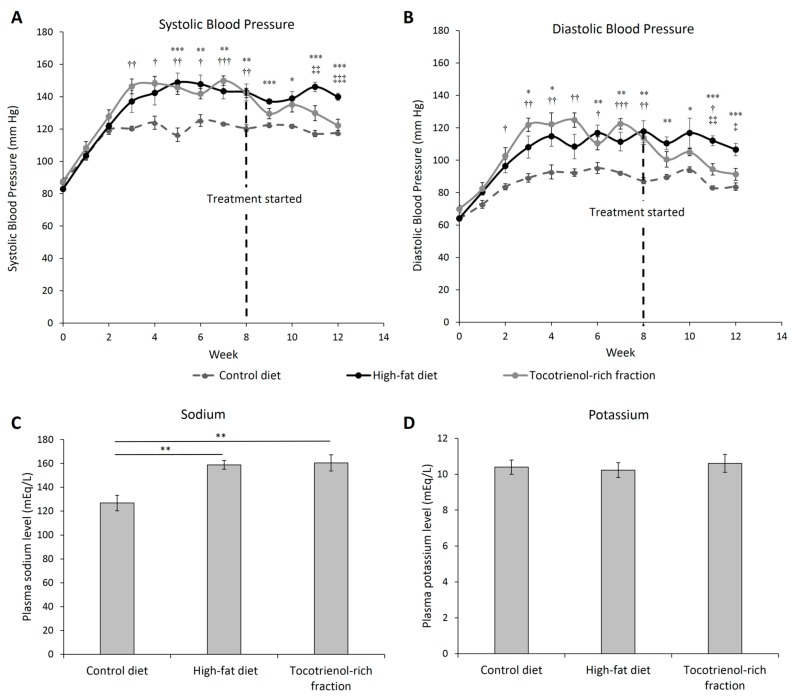
Systolic (**A**) and diastolic (**B**) blood pressure of the rats assigned to different treatment groups throughout 12 weeks. A tocotrienol-rich fraction was administered after 8 weeks of high-fat feeding as indicated by the black dotted line. Plasma sodium (**C**) and potassium (**D**) levels at the end of treatment was measured. Error bars indicate SEM. The sample size was *n* = 7 per group. For Figure 3A,B, *, **, *** indicate *p* < 0.05, 0.01, 0.001 between and control and high-fat diet groups; †, ††, ††† indicate *p* < 0.05, 0.01, 0.001 between the control diet and tocotrienol-rich fraction groups; ‡, ‡‡, ‡‡‡ indicate *p* < 0.05, 0.01, 0.001 between the high-fat diet and tocotrienol-rich fraction groups. For Figure 3C, ** Indicates *p* < 0.01 between groups.

Aside from central obesity, high-fat feeding caused a significant increase in systolic blood pressure compared to the control diet from Week 4 onwards (Figure 3A). The diastolic blood pressure followed a similar trend (Figure 3B). The hypertension was linked to increased fluid retention as indicated by the high-fat-diet-induced hypernatremia (158.74 ± 3.64 mEq/L vs. 126.73 ± 6.58 mEq/L) (Figure 3C). Interestingly, the TRF lowered the systolic and diastolic blood pressure gradually over four weeks. The difference in blood pressure between high-fat-diet- and TRF-treated groups became significantly different two weeks into treatment. Such an antihypertensive effect of the TRF seems to be independent from the water reabsorption mechanism as the elevated sodium level was not improved. Previous studies have suggested that the blood pressure-lowering effect may be linked to vitamin E-induced vasodilation [17,30]. The potassium level was not affected by the diets or the TRF (Figure 3D).

### 3.3. Glycemic Parameters and OGTT

**Figure 4:**
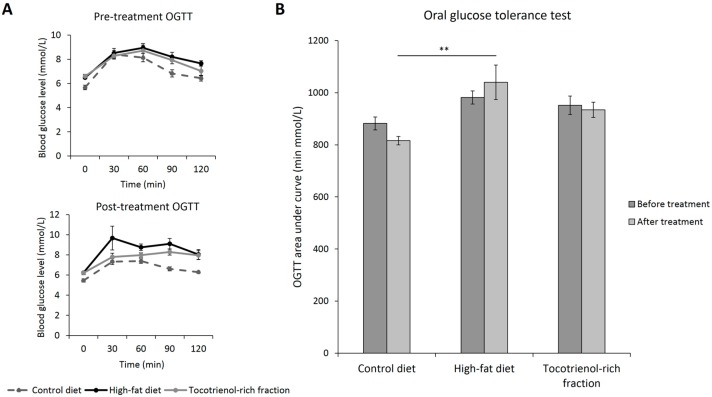
Oral glucose tolerance test before (Week 8) and after (Week 12) treatment (**A**). The AUCs are expressed in bar plot (**B**). Error bars indicate SEM. The sample size was *n* = 7 per group. ** Indicates *p* < 0.01 between groups. OGTT: oral glucose tolerance test.

Based on Table 3, high-fat consumption induced polydipsia, increased fasting blood glucose, and impaired β-cell function in addition to the impaired glucose tolerance, as shown in Figure 4A,B. These are the typical symptoms of diabetes mellitus. Treatment with the TRF failed to improve these abnormalities, suggesting that the fraction has minimal impact on glucose homeostasis.

### 3.4. Oxidative Stress Markers and AGE-RAGE Axis

**Figure 5:**
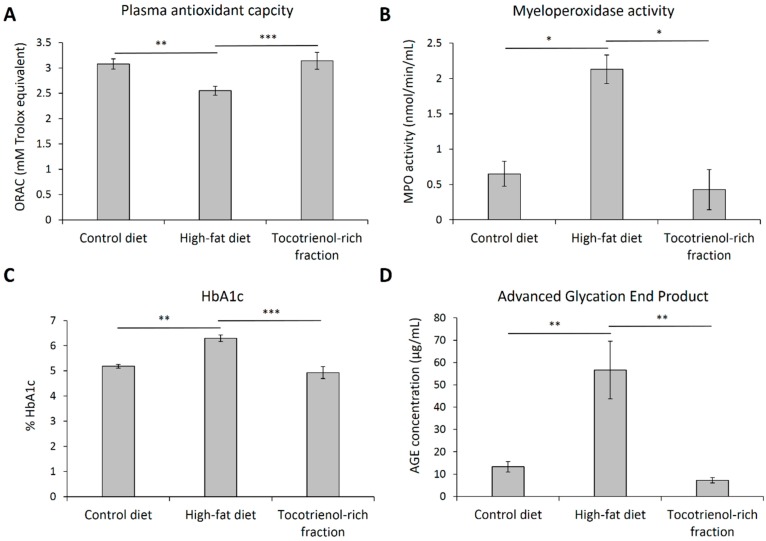
Total plasma antioxidant capacity (**A**), myeloperoxidase activity (**B**), glycated hemoglobin A1c (**C**), and advanced glycation end product (**D**) of the rats assigned to different treatment groups at the end of treatment. Error bars indicate SEM. The sample size was *n* = 7 per group. * Indicates *p* < 0.05, ** Indicates *p* < 0.01, and *** Indicates *p* < 0.001 between groups. AGE: advanced glycation end product; HbA1c: glycated hemoglobin A1c; MPO: myeloperoxidase; ORAC: oxygen radical absorbance capacity.

Given that tocotrienols are strong antioxidants, we also examined the several oxidative stress biomarkers at the end of the experiment. It was revealed that the TRF was able to recover the plasma antioxidant capacity, which was otherwise crippled by the high-fat feeding by about 17% (Figure 5A). Furthermore, the activity of myeloperoxidase, a peroxidase enzyme that can promote oxidative stress, was elevated by more than twofold upon high-fat consumption. Such an exaggerated activation of myeloperoxidase was abolished with the treatment of the TRF (Figure 5B), which is in line with the plasma antioxidant capacity assay.

The TRF also successfully reduced protein glycation, as evidenced by the lowering effect on HbA1c (Figure 5C) and circulating AGE (Figure 5D). Although non-enzymatic glycation is a glucose-dependent process, the anti-glycative effect of the TRF seems to be glucose-independent because the treatment did not have a noticeable effect on glucose metabolism. It is postulated that the effect may be attributable to its antioxidant activity, as high oxidative stress could have otherwise promoted the formation of glycation products.

**Figure 6:**
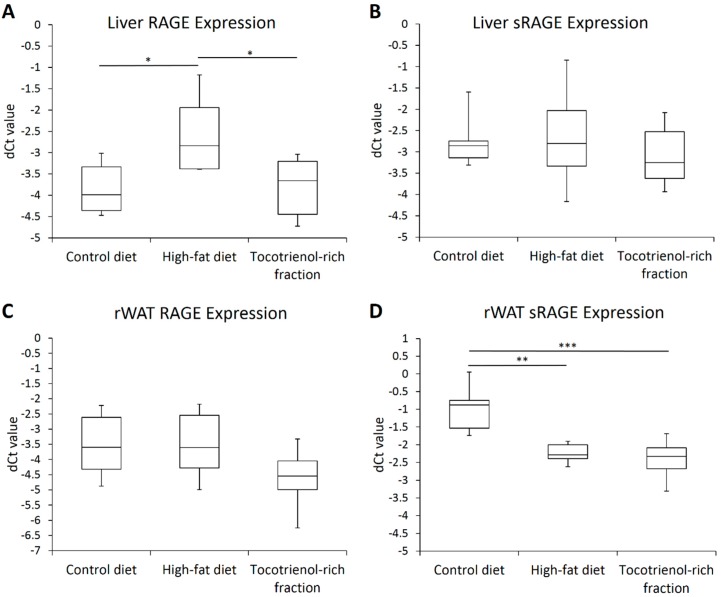
Box-and-whisker plots of normalized Ct values (dCt) of RAGE and sRAGE in the liver (**A**,**B**) as well as retroperitoneal adipose tissue (**C**,**D**) of the rats assigned to different treatment groups. Hypoxanthine phosphoribosyltransferase 1 (HPRT1), succinate dehydrogenase complex flavoprotein subunit A (SDHA), and β-actin (BAC) were used as the endogenous reference genes. Sample size was *n* = 7 per group. * Indicates *p* < 0.05, ** Indicates *p* < 0.01, and *** Indicates *p* < 0.001 between groups. RAGE: receptor for advanced glycation end product; sRAGE: soluble receptor for glycation end product; rWAT: retroperitoneal white adipose tissue.

Additionally, considering such a drastic escalation of the circulating AGE level upon high-fat feeding and the AGE-lowering effect of the TRF, we endeavored to determine whether or not these changes modified the expression of RAGE in the liver and rWAT. Expectedly, hepatic RAGE expression was significantly upregulated 2.4-fold in the high-fat diet group. RAGE overexpression was nullified by the treatment with TRF, which is in concordance to the circulating AGE level (Figure 6A). RAGE expression in the rWAT was unaffected by the types of diet and the TRF (Figure 6C). 

Apart from that, high-fat consumption also suppressed the expression of sRAGE in the rWAT 2.3-fold, which might lead to lower circulating sRAGE and consequently increased AGE accumulation. The TRF did not improve the downregulation of sRAGE (Figure 6D). On the other hand, neither the diets nor the TRF modified sRAGE expression in the liver (Figure 6B). The differential RAGE and sRAGE expression patterns between the liver and rWAT are indicative of a certain extent of tissue specificity in the expression of RAGE and its spliced variants.

### 3.5. Lipid Profile, Hepatic Steatosis, and PPAR Expression

**Figure 7:**
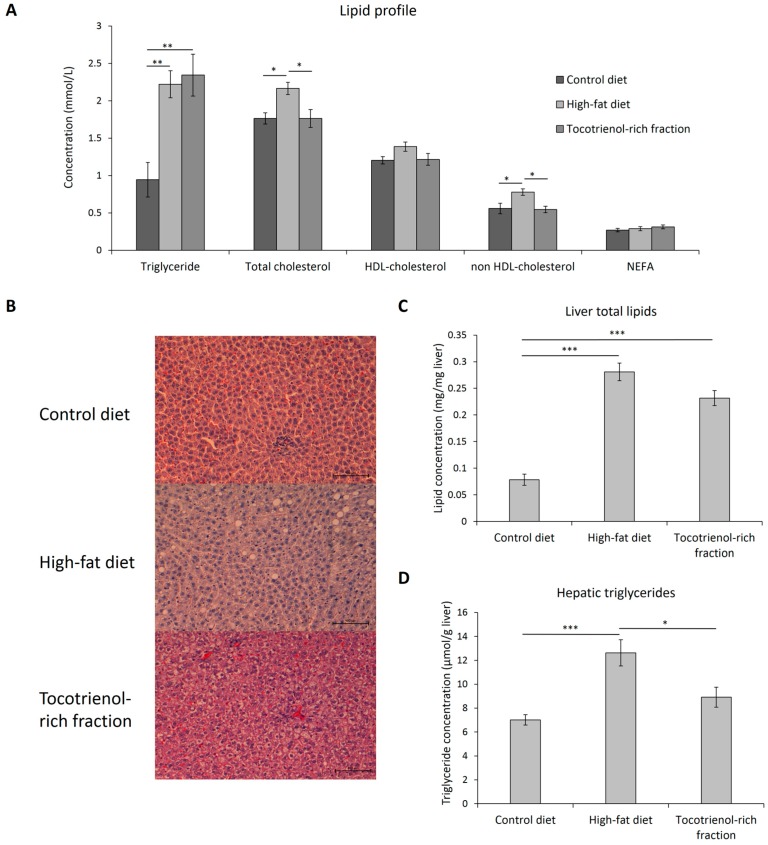
Lipid profile (**A**), including triglycerides, total cholesterol, HDL-cholesterol, non HDL-cholesterol and non-esterified free fatty acid levels of the rats assigned to different treatment groups at the end of treatment. The representative images of the H&E-stained liver tissue (x200 magnification) of each group are shown (**B**). The total lipids (**C**) and triglycerides (**D**) concentrations in the liver were quantified and illustrated in bar plots. Error bars indicate SEM. The sample size was *n* = 7 per group. * Indicates *p* < 0.05, ** Indicates *p* < 0.01 and *** Indicates *p* < 0.001 between groups. HDL, high density lipoprotein; NEFA, non-esterified fatty acid.

Treatment with the TRF also reversed the high-fat-diet-induced hypercholesterolemia (Figure 7A). The total cholesterol and non HDL-cholesterol levels were reduced by the TRF by 19% and 29% respectively after the four-week treatment. However, the circulating triglyceride level remained elevated despite the administration of the TRF, indicating that the treatment is more specific on the cholesterol metabolism. Additionally, consumption of high-fat diet also triggered increased ectopic fat deposition in the liver (Figure 7B). Compared high-fat-diet-treated rats to those on control diet, the liver total lipids increased 3.6-fold from 0.08 ± 0.01 mg/mg liver to 0.28 ± 0.02 mg/mg liver whereas the hepatic triglycerides deposition also escalated from 7.02 ± 0.43 μmol/g liver to 12.63 ± 1.10 μmol/g liver. Treatment with the TRF did not lead to significant reduction of the total lipids (0.23 ± 0.01 mg/mg liver; *p* = 0.063), but did significantly reduce the triglycerides deposition to 8.92 ± 0.84 μmol/g liver (Figure 7C,D). 

**Figure 8:**
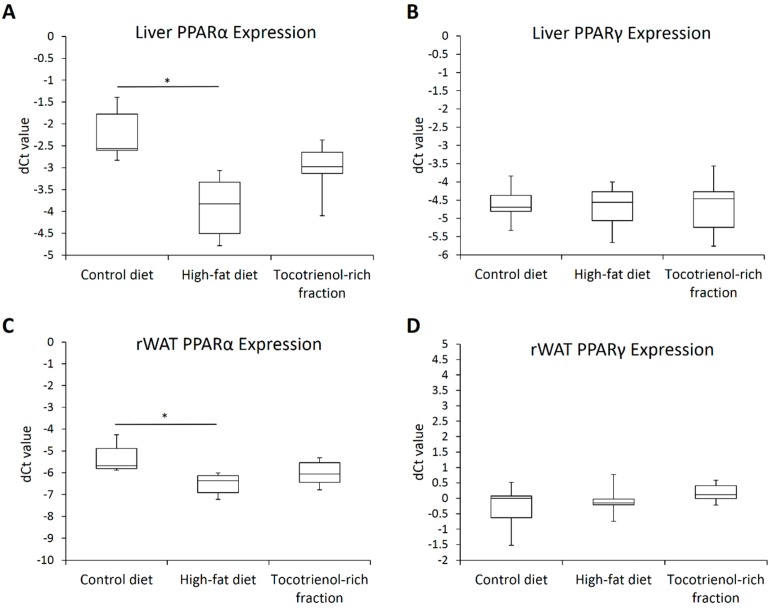
Box-and-whisker plots of normalized Ct values (dCt) of PPARα and PPARγ in the liver (**A**,**B**) as well as retroperitoneal adipose tissue (**C**,**D**) of the rats assigned to different treatment groups. Hypoxanthine phosphoribosyltransferase 1 (HPRT1), succinate dehydrogenase complex flavoprotein subunit A (SDHA), and β-actin (BAC) were used as the endogenous reference genes. Sample size was *n* = 7 per group. * Indicates *p* < 0.05 between groups. PPAR: peroxisome proliferator-activated receptor; rWAT: retroperitoneal white adipose tissue.

The results showed that the TRF has inhibitory effects on the lipid dysregulation. Since PPARs are the key regulators of lipid metabolism and adipogenesis, we also looked into the expression of PPARα and γ in our attempt to outline the underlying mechanism of the TRF. Basically, high-fat feeding suppressed the expression of PPARα in both the liver and rWAT 3.1- and 2.3-fold, respectively (Figure 8A,C). Transcriptional suppression of PPARα was not reversed by the TRF. The expression of PPARγ was unremarkable (Figure 8B,D). 

## 4. Discussion

In the present study, we demonstrated that a TRF can confer multiple beneficial effects on the rats on high-fat diet. After a four-week treatment of the fraction via oral administration at a daily dosage of 60 mg/kg, the rats showed significant improvements in terms of the blood pressure, cholesterol profile, ectopic fat deposition at the liver, and oxidative stress-related markers. Despite the unremarkable effect on glycemic control and central obesity, such multifunctionality of a TRF against metabolic syndrome still makes it an interesting candidate for further investigation and potential clinical use.

Our results pointed out that a TRF possesses remarkable antihypertensive activity. This is consistent with previous animal [17] and clinical studies [31]. Nevertheless, the effect is not exclusive to tocotrienol, but is a common property of vitamin E as evidenced by the comparable hypotensive effect of both α-tocopherol and α-tocopherol-tocotrienol mixture [17,32]. In this study, the diet-induced hypertension was linked to hypernatremia. This is in line with a previous study that indicated that a high-fat diet can promote sodium and water retention [33]. However, the TRF failed to reverse the elevated sodium level, implying that the antihypertensive effect is independent of the water and sodium reabsorption pathway. In fact, as the blood pressure-lowering activity is a shared characteristic between tocotrienols and tocopherols, the underlying mechanism should also be mutual. Previous studies have reported that vitamin E can alleviate vascular oxidative stress and stimulate the aortic biosynthesis of prostacyclin, which has in turn resulted in vasodilation [17,30,34]. Thus, it is postulated that the antihypertensive effect of vitamin E is attributable to antioxidant-dependent vasodilation.

Although several studies have reported the glucose-lowering effect of tocotrienol [13,14,35,36], such an effect was not noticeable in the present study. It is worth mentioning that most of the aforementioned studies employed streptozotocin-induced diabetic rats whose hyperglycemia induction is dependent on the toxicity effect of streptozotocin on pancreatic β-cells. Tocotrienols have been shown to confer localized ameliorative effect on streptozotocin-induced cellular damage at the cerebral tissues by alleviating oxidative-nitrosative stress [37]. Furthermore, many studies that have reported the antihyperglycemic effect have either used tocotrienols pre-treatment prior to [35] or considerably fast treatment (~3 days) following [14,15,16] a streptozotocin challenge. This means that tocotrienols were introduced while the β-cells destruction caused by streptozotocin was still ongoing. In this context, tocotrienols are capable of reducing oxidative DNA damage [13,38,39]. Speculatively, this may protect the pancreatic β-cells from the insult of streptozotocin, which may in turn lead to a favorable glycemic profile. This may explain why we failed to detect improved glycemic control upon treatment with the TRF because extensive eradication of pancreatic β-cells, as is induced by streptozotocin, is uncommon in metabolic syndrome. Nonetheless, to our best knowledge, no clinical studies have demonstrated the glucose-lowering effect of tocotrienols in diabetic patients; hence, further investigation on this aspect is warranted. Future studies using streptozotocin-induced diabetic models should also evaluate the possible anti-streptozotocin activity of tocotrienols to eliminate the interference from the actual glucose-lowering effect.

As a powerful lipophilic antioxidant, treatment with the TRF also restored the total plasma antioxidant capacity in addition to abolishing myeloperoxidase hyperactivity. These findings show that the TRF could alleviate the oxidative stress in the systemic level [15,16]. Given that myeloperoxidase is highly implicated in atherogenesis [40,41], supplementation with a TRF could therefore prevent the development of atherosclerosis [42,43]. Additionally, a TRF also possesses anti-glycative activity, as has been shown by the reduction of HbA1c and AGE [35]. Although non-enzymatic glycation is thought to be a glucose-dependent process, it has long been known that increased oxidative stress could accelerate non-enzymatic glycation and promote the accumulation of glycated proteins and AGE precursors [44]. In the present study, the failure to reverse glycemic control and sRAGE expression points out that the TRF did not act on the glucose-dependent processes in the protein glycation as well as AGE detoxification, respectively. Therefore, such an anti-glycative activity of the fraction should be antioxidant-dependent [45]. Apart from that, we also demonstrated, for the first time, that the TRF could inhibit diet-induced transcriptional activation of RAGE in the liver. Such an AGE-RAGE inhibitory effect is complementary to its modulatory effect on the nuclear factor κB signaling cascade and certain proinflammatory cytokines [46]. Thus, the use of a TRF could potentially alleviate the proinflammatory response and potentiate the effects of other therapeutic agents in the treatment of metabolic syndrome and diabetes mellitus.

Next, treatment with the TRF also reversed the high-fat-diet-induced hypercholesterolemia by lowering total cholesterol and non-HDL cholesterol levels, which is in line with several clinical trials [19,20,21]. The cholesterol-lowering effect is due to the inhibitory effect of tocotrienols on β-hydroxy-β-methylglutaryl coenzyme A (HMG-CoA) reductase [7,47]. The normalized cholesterol profile also brought about significant improvement to hepatic steatosis. To date, the evidence for the hepato-protective effect of tocotrienols against diet-induced fatty liver is still limited. Two studies have been conducted using high-calorie diet-fed rats and hypercholesterolemic patients, respectively [36,48], both of which revealed a favorable effect of the TRF on hepatic lipid deposition. Mechanistically, high-fat feeding triggers the overexpression and hyperactivity of sterol regulatory element binding protein (SREBP)-2 and HMG-CoA reductase in the liver [49]. These abnormalities are effectively abolished by tocotrienols and thus contribute to hepato-protective activity [47,50]. 

Furthermore, high-fat feeding induced the transcriptional repression of PPARα in the liver and rWAT, which could also promote ectopic fat deposition because of the key regulatory roles of PPARα in fatty acid β-oxidation [51]. The effect of the TRF on PPARα expression was unremarkable. Likewise, there was no change in the expression of hepatic and rWAT PPARγ. This points out that the ameliorative effects of the TRF on abnormal cholesterol profile and hepatic steatosis were PPAR-independent. In this case, even though the PPAR agonistic activity of tocotrienols has been demonstrated, it is established primarily through cell culture studies [43,52]. When the muscle tissues of TRF-treated mice were examined, there was no noticeable change in the expression of PPARs [52]. Together with the lack of PPAR activation in the liver and rWAT, in which PPARs are highly expressed, it is believed that in vivo PPAR agonistic activity of tocotrienols is marginal. 

Lastly, our study was limited by the use of a TRF that contains 23.5% (*w*/*w*) of α-tocopherol. Therefore, possible interaction of the α-tocopherol cannot be excluded. Furthermore, we were also unable to differentiate the individual bioactivities of the four tocotrienol subtypes. With regard to the inhibition of HMG-CoA reductase, γ-tocotrienol is 30 times more effective than α- and δ-tocotrienols [53]. This signifies possible differences in the biological functionality of different tocotrienol isomers [54]. Nonetheless, this study, in general, is concordant with most clinical findings on diabetic and hypercholesterolemic patients [19,21] that show that tocotrienols have potent inhibitory effects on hypertension, hypercholesterolemia, and elevated oxidative stress, but have marginal effects on weight control and glucose metabolism. These beneficial effects support further study and the possible clinical use of a TRF as therapy for metabolic syndrome. 

## 5. Conclusions

In conclusion, treatment with a TRF from palm oil for four weeks in rats with metabolic syndrome showed significant improvements in blood pressure, cholesterol profile, and systemic antioxidant defense in addition to a reduction in hepatic steatosis, proatherogenic markers such as myeloperoxidase, and proinflammatory markers such as advanced glycation end products. It was also demonstrated for the first time that a TRF can confer an inhibitory effect on RAGE transcriptional activation. Unlike as reported in previous studies, the treatment had minimal impact on glucose metabolism, PPAR expression, hypertriglyceridemia, and visceral adiposity. Further investigation on these aspects is warranted. As such, in light of the beneficial effects on cardiovascular health, lipid metabolism, redox balance, and anti-inflammation, a TRF is undoubtedly a promising candidate for metabolic syndrome therapy. 

## Figures and Tables

**Table 1:** Macronutrient composition and ingredients of control and high-fat diets.

Macronutrient	Control Diet	High-fat Diet
Protein (kcal %)	20	20
Carbohydrate (kcal %)	70	20
Lipid (kcal %)	10	60
Saturated (%)	36.6	57.9
Monounsaturated (%)	29.0	28.8
Polyunsaturated (%)	32.0	8.4
Trans (%)	1.8	3.6
Energy content (kcal/g)	3.9	5.3
Ingredient	Mass (g)	
Casein	200	200
L-cystine	3	3
Corn starch	525.5	18
Maltodextrin	125	125
Sugar	50	50
Cellulose	50	50
Milk fat	20	245
Corn oil	25	25
AIN-93G Mineral mix	35	35
AIN-93-VX Vitamin mix	10	10
Vitamin A	0.016	0.016
Vitamin B	0.092	0.092
Vitamin D	0.003	0.003
Vitamin E (α-tocopherol)	0.3	0.3
Vitamin K	0.001	0.001
Choline bitartrate	2	2
t-butylhydroquinone	0.014	0.014

**Table 2:** Accession numbers, forward and reverse primers of the endogenous reference, and target genes as well as amplicon size of the PCR products.

Target Gene	Accession Number	Nucleotide Sequence (5′→3′)		Amplicon Size (bp)
		Forward Primer	Reverse Primer	
β-actin *	NM_031144	GTA TGG GTC AGA AGG ACT CC	GTT CAA TGG GGT ACT TCA GG	80
HPRT1 *	NM_012583	CTG GAA AGA ACG TCT TGA TTG	GTA TCC AAC ACT TCG AGA GG	146
SDHA *	NM_130428	GGC TTT CAC TTC TCT GTT GG	CCA CAG CAT CAA ACT CAT GG	103
RAGE	NM_053336	CGA GTC TAC CAG ATT CCT GGG	TCA CAA CTG TCC CTT TGC CA	175
sRAGE	GUI164719	CAA TGT CCC CTG CCT CCA GA	TCA TCC TCA TGC CCT ACC TCA	200
PPARα	NM_013196	TGT GGA GAT CGG CCT GGC CTT	CCG GAT GGT TGC TCT GCA GGT	100
PPARγ	NM_013124	CCC TGG CAA AGC ATT TGT AT	GGT GAT TTG TCT GTT GTC TTT CC	100

* Endogenous reference genes; HPRT1: Hypoxanthine phosphoribosyltransferase 1; PPAR: peroxisome proliferator-activated receptor; RAGE: receptor for advanced glycation end product; SDHA: succinate dehydrogenase complex flavoprotein subunit A; sRAGE: soluble receptor for advanced glycation end product.

**Table 3:** Food and water intake, organ weight, and glycemic indices of the rats given the control diet, the high-fat diet, and the TRF at the end of the eight-week treatment.

Parameters	Treatment Group		
	Control Diet	High-Fat Diet	Tocotrienol-Rich Fraction
Water intake (mL/day)	18.97 ± 1.07	25.14 ± 1.35 **	21.46 ± 0.99
Liver-to-body weight ratio (%)	3.35 ± 0.05	3.72 ± 0.13	3.71 ± 0.16
Kidney-to-body weight ratio (%)	0.73 ± 0.02	0.74 ± 0.01	0.73 ± 0.02
FBG (mmol/L)	5.51 ± 0.15	6.50 ± 0.14 **	6.27 ± 0.17 **
FPI (mU/L)	28.23 ± 2.27	25.54 ± 2.13	24.84 ± 1.74
HOMA%β (%)	100.00 ± 7.51	67.71 ± 5.89 **	71.11 ± 5.42 *
HOMA%S (%)	100.00 ± 6.61	106.32 ± 8.10	109.52 ± 6.88

Values are expressed as mean ± SEM; AUC: area under curve; FBG: fasting blood glucose; FPI: fasting plasma insulin; HOMA%S: homeostasis model assessment of insulin sensitivity; HOMA%β: homeostasis model assessment of β-cell function. * *p* < 0.05, and ** *p* < 0.01 compared to the control diet.

## Supplementary Materials

The following are available online at www.mdpi.com/2072-6643/9/9/984/s1: Table S1: Quantitative PCR condition of the endogenous reference and target genes.
