# Supplementary material for: The Ameliorative Effects of a Tocotrienol-Rich Fraction on the AGE-RAGE Axis and Hypertension in High-Fat-Diet-Fed Rats with Metabolic Syndrome
Source: Nutrients. 2017 Sep 7;9(9):984. doi: 10.3390/nu9090984 (PMC5622744; doi:10.3390/nu9090984)
Supplement: Supplementary file 1 [file nutrients-09-00984-s001.pdf]

Supplementary Materials

Table S1. Quantitative PCR condition of the endogenous reference and target genes.

Target gene	Initial denaturation	Denaturation	Annealing	Elongation
		(cycle for 40 times)		
β-actin*			48°C, 30 s	
HPRT1*			47°C, 30 s	
SDHA*			52°C, 30 s	
RAGE	94°C, 2 mins	94°C, 15 s	53°C, 30 s	72°C, 30s
sRAGE			58°C, 30 s	
PPARα			66°C, 30 s	
PPARγ			54°C, 30 s	

* endogenous reference genes; HPRT1: hypoxanthine phosphoribosyltransferase 1; PPAR: peroxisome proliferator-activated receptor; RAGE: receptor for advanced glycation end product; SDHA: succinate dehydrogenase complex flavoprotein subunit A; sRAGE: soluble receptor for advanced glycation end product.
